# Eye health promotion in multi-sectoral settings: A systematic review of intervention types and effectiveness

**DOI:** 10.12688/f1000research.168998.1

**Published:** 2025-09-18

**Authors:** Hlabje Carel Masemola, Sphamandla Josis Nkambule, Olivia Baloyi, Zamadonda Nokuthula Xulu-Kasaba

**Affiliations:** 1 Department of Public Health Medicine, School of Nursing and Public Health, College of Health Sciences, University of KwaZulu-Natal, Durban, Kwazulu Natal, 4041, South Africa; 2 Department of Public Health Medicine, School of Nursing and Public Health, College of Health Sciences, University of KwaZulu-Natal, Durban, Kwazulu Natal, 4041, South Africa; 3 Department of Nursing, Walter Sisulu University, Umtata, Eastern Cape, 5099, South Africa; 4 Department of Optometry, School of Nursing and Public Health, College of Health Sciences, University of KwaZulu-Natal, Durban, KwaZulu Natal, 4041, South Africa

**Keywords:** Eye health, health promotion, multi-sectoral approach, public health interventions, primary eye care, health systems

## Abstract

**Background:**

Multi-sectoral settings such as hospitals, health care centres, clinics, schools, workplaces, community events, and online platforms play an important role in reaching individuals from various socioeconomic backgrounds and delivering health promotion interventions that change behaviour and improve health outcomes. The aim of this review was to systematically review the nature and effectiveness of eye health promotion interventions implemented by eye health professionals across various multi-sectoral settings.

**Method:**

This review was conducted using the Centre for Review and Dissemination guidelines for conducting systematic reviews and reported in accordance with the PRISMA 2020 statement. A comprehensive and systematic search was conducted on electronic databases such as PubMed, Web of Science, and Medline, CINAHL and Academic Search Complete hosted on EBSCOHost and supplemented with a hand search for relevant studies published between 1999 and 2024. Two independent reviewers screened eligible studies, extracted data, and assessed study quality using the Mixed Methods Appraisal Tool (MMAT) 2018. The eligibility criterion was met by 80 studies out of 15 554 screened.

**Results:**

Eye health promotion interventions identified among studies were categorised as health education, health counselling, vision screening, eye screening and training programmes, health prevention, health promotion, health policy, ophthalmic intervention programmes, comprehensive eye examination and screening for diabetic retinopathy. Most studies showed a significant impact of eye health promotion interventions.

**Conclusion:**

Overall, studies demonstrated a diverse range of strategies that have shown significant effectiveness across various settings. This systematic review highlights the effectiveness of eye health promotion interventions, whilst identifying gaps in compliance, follow-up systems, and policy integration.

Systematic review registration PROSPERO CRD42022354299.

## Introduction

Multi-sectoral settings such as hospitals, clinics, schools, community events and online platforms play an important role in reaching individuals from various socioeconomic backgrounds. Additionally, these settings improve the delivery of health promotion interventions to change behaviour and improve health outcomes. While health promotion initiatives are traditionally delivered within clinical and healthcare settings, there is a growing recognition of the importance of implementing eye health promotion initiatives in multi-sectoral settings. ^1^ Evidence suggests that targeting communities or populations, rather than individuals, through health promotion initiatives, usually has significant cross-sectoral effects and financial ramifications. ^2^ The significance of eye health promotion arises from the global burden of vision impairment (VI) and blindness, which not only affects individuals’ quality of life but also poses substantial economic and social challenges. ^3^

Eye health promotion interventions play a pivotal role in preventing VI and promoting overall ocular well-being across diverse populations. ^4^ These interventions encompass a spectrum of activities aimed at raising awareness, encouraging preventative behaviours, facilitating early detection, and ensuring access to appropriate eye care services. ^5^ As per the World Health Organization (WHO), uncorrected refractive errors and cataracts are the primary causes of VI and blindness, affecting an estimated 2.2 billion people globally. ^6^ Moreover, the prevalence of VI is expected to rise due to ageing populations, lifestyle changes, and the increasing prevalence of chronic diseases such as diabetes, which can lead to diabetic retinopathy and other vision-threatening conditions. ^7^ The integration and alignment of eye health promotion strategies can reduce the impact of this burden of VI and improve health. ^6^

Despite efforts to promote health over the past four decades, evidence suggests that a large part of health expenditure is spent on biomedical care, limiting the success of health promotion activities. ^8– 10^ The establishment of effective health promotion and prevention campaigns such as behavioural approaches and screenings, including door-to-door interventions, national screening campaigns, educational programmes and mobile screening strategies, have the potential to be effective in low-and middle-income countries (LMICs). ^11, 12^ In clinical and healthcare settings, studies have demonstrated the efficacy of interventions such as health literacy, patient education on healthy lifestyle behaviours, and provision of subsidized or free eye screenings in improving awareness and adherence to preventive eye care measures. ^13– 15^ These interventions not only facilitate early detection of eye conditions but also promote timely access to treatment, ultimately contributing to better visual outcomes and patient satisfaction.

In educational settings, particularly schools, research has shown the positive impact of vision screening programs and health education initiatives on children’s eye health. ^16^ By integrating vision screening into school health programs and providing educational materials on eye care, schools can identify students with ocular problems early and facilitate their access to follow-up care, including prescription eyewear and treatment for underlying eye conditions. Moreover, promoting eye health awareness in schools can empower students to adopt healthy behaviours and advocate for their ocular well-being, fostering a culture of proactive eye care from a young age.

Workplaces, by virtue of their social network presence, offer large populations who spend extensive amounts of time at work, making them a rewarding setting for health promotion. ^17^ Moreover, workplace programmes focusing on improving health-related behaviours such as diet, physical activity and tobacco use are largely effective when evidence-based interventions are used. ^18^ Workplace-based interventions have drawn interest due to their ability to preserve employees’ eyes from current health risks, limiting exposure to specific occupational hazards and offering an option to account for workers’ natural sight loss in risk assessments. ^19^ Occupational safety programs, ergonomic assessments, and the provision of protective eyewear in high-risk industries have been shown to reduce the incidence of work-related eye injuries and promote a safe working environment. ^20^ Additionally, employee wellness initiatives that incorporate behavioural interventions, workplace environmental controls, and primary ocular care health services like vision screenings, eye health education, and the use of various eye protection devices have the potential to lower healthcare costs for employers whilst increasing productivity. ^21^

While considerable efforts have been made to address VI through medical and surgical interventions, the importance of preventive strategies cannot be overemphasised. Eye health promotion interventions, including education, early detection, and access to eye care services, are cost-effective approaches for reducing the burden of VI. By promoting healthy behaviours, encouraging regular eye examinations, and ensuring timely interventions, these initiatives have the potential to prevent or delay the onset of vision loss and its associated complications. However, the effectiveness of these interventions in multi-sectoral settings remains an area of interest and requires further investigation to inform evidence-based practices and policies.

Despite the growing recognition of the importance of multi-sectoral approaches to eye health promotion, there is limited comprehensive evidence on the effectiveness of interventions delivered in various settings outside the traditional healthcare environment. Addressing this gap is crucial for optimizing resource allocation, designing tailored interventions, and maximizing the impact of eye health promotion efforts. Therefore, this systematic review aims to synthesize existing evidence on the nature and effectiveness of eye health promotion interventions implemented in multi-sectoral settings. By examining the outcomes, methodologies, and contextual factors associated with these interventions, this study seeks to provide insights that can guide the implementation of eye health promotion interventions.

## Materials and methods

This systematic review was designed following the Preferred Reporting Items for Systematic Reviews and Meta-Analyses Protocol Extension (PRISMA-P) (S1 Checklist). ^22^ The protocol has been registered with the PROSPERO (CRD42022354299) and published in the Plos One journal. ^23^ This systematic review followed the Center for Reviews and Dissemination’s (CRD) guidelines for conducting systematic reviews in healthcare. ^24^

This study addressed the following research questions:

1. What is the nature of eye health promotion interventions implemented by eye care professionals in multi-sectoral contexts; and
2. What is the effectiveness of eye health promotion interventions implemented by eye care professionals in multi-sectoral contexts?

### Search strategy

A systematic literature search was conducted following the Centre for Reviews and Dissemination (CRD) guidance and reported following the PRISMA statement. The search targeted studies on the nature and effectiveness of eye health promotion interventions. Electronic databases were searched: PubMed, Web of Science, and Medline, CINAHL and Academic Search Complete hosted on EBSCOHost. Searches were conducted from the inception of the Vision 2020 Campaign (1999) to November 2024. The search strategy included a combination of Medical Subject Headings (MeSH) and free-text terms related to “eye health promotion,” “vision care,” “health interventions,” “public health campaigns,” and “visual impairment prevention.” Supplementary searches involved hand-searching in relevant journals, reviewing reference lists of identified studies. (refer to underlying data: S1 Table). ^22^

### Inclusion and exclusion criteria

Studies were included if they met the following criteria: (1) peer-reviewed primary research studies (randomized controlled trials, quasi-experimental studies, observational studies, and qualitative studies); (2) focused on eye health promotion interventions aligned with the Ottawa Charter for Health Promotion action areas; (3) reported on outcomes such as improved access to eye care, behaviour change, visual acuity, prevention of blindness, or policy changes. Studies were excluded if they (1) did not address eye health promotion, (2) were systematic reviews or meta-analyses, (3) were published only as abstracts with insufficient information, or (4) were non-English publications.

### Study selection

The selection process followed PRISMA 2020 guidelines. All records retrieved from the searches were imported into EndNote X9 for reference management, and duplicates were removed. Two reviewers (HCM and SJN) independently screened the titles and abstracts against the inclusion criteria. Full-text articles of potentially relevant studies were retrieved and assessed independently by the same reviewers. Discrepancies were resolved through discussion or consultation with a third reviewer.

### Data extraction

Data extraction followed the Template for Intervention Description and Replication (TIDieR) checklist to ensure detailed reporting of interventions. A standardized data extraction tool was created as a link on forms and piloted on three studies to ensure accuracy and consistency. Thereafter, it was finalised for use in the study.

Extracted data included study characteristics (authors, year, setting, design), participant demographics, intervention details (type, duration, delivery method), and outcomes. Quantitative data were extracted using a double entry approach where two independent reviewers entered it onto a previously piloted tool.

### Quality assessment

The methodological quality of all included studies was assessed using the Mixed Methods Appraisal Tool (MMAT) 2018 due to the included studies using differing or multiple methodologies. Two reviewers (HCM and SJN) independently appraised the quality each empirical studies across five criteria. Any disagreements were resolved through discussion with a third researcher. The checklist’s first two questions, which are the same for all study designs, evaluate whether the research questions are clearly stated and whether the data gathered allows them to be addressed. Based on the relevant study design, reviewers answer five distinct questions for each type of study. The possible answers are “Yes,” “No,” and “can’t tell.” A study’s score on the quality assessment tool is not an indicator in determining whether or not a study is included in the review. (refer to underlying data: S2 Table). ^22^

### Synthesis, summarising and reporting of results

Collation of the findings from this systematic review was guided by an eye health promotion intervention, and whether the articles assessed the nature or effectiveness of that intervention. Results were summarized using descriptive numerical descriptions of the data. A summary of findings was tabled, highlighting key outcomes, study characteristics, and quality assessments. The Ottawa Charter Framework was used as a framework to categorize and interpret the findings. Due to high heterogeneity in intervention types, populations, and outcome measures, meta-analysis was not feasible; therefore, a descriptive synthesis was conducted.

## Results

### Study selection

**Figure 1.**
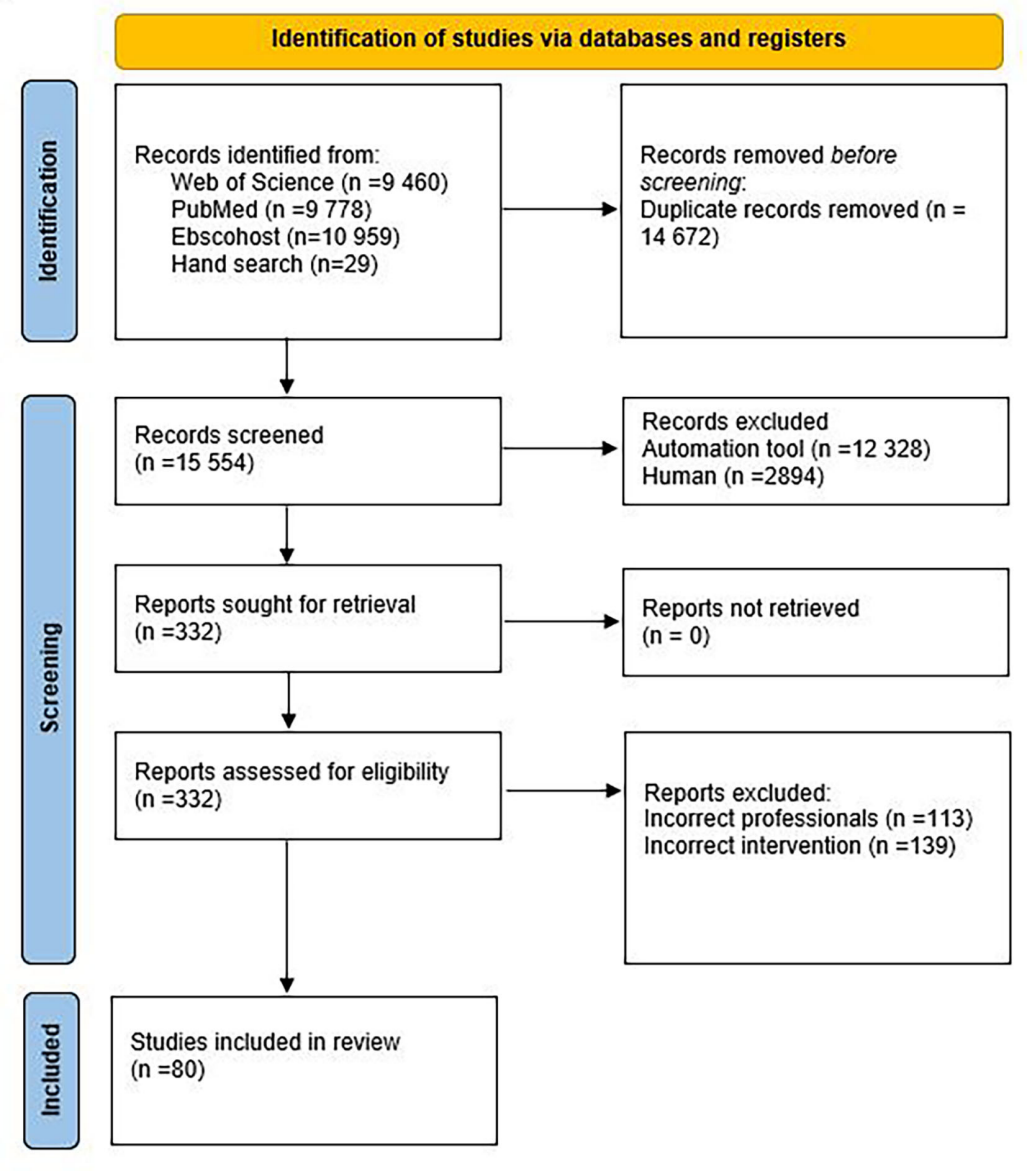
Flow diagram of the screening process according to the PRISMA.

The initial search resulted in 15 554 citations after removing 14 672 duplicates ( Figure 1). ^22^ During title and abstract screening, 15 132 citations that did not meet the inclusion criteria, and were excluded yielding 332 citations eligible for full-text screening. After close examination during full-text screening, 133 citations were excluded as they were reporting on interventions conducted by non-eye health professionals and 139 citations reported on interventions that were not related to health promotion. Ultimately, 80 citations met the inclusion/exclusion criteria and were included in the systematic review synthesis.

### Study characteristics

The summary of the included studies and their characteristics such as study title, study aim, sample size, type of intervention, tools of measurement and other pertinent information is shown in S3 Table- refer to underlying data. ^22^ Studies included in this systematic review consist of different research designs, reflecting a diverse approach to answering the research question. Concerning study designs, a total of 21 cross-sectional, ^25– 45^ 18 cohort, ^46– 63^ and nine randomised control trials ^64– 72^ were included for analysis. Nine studies employed quantitative telemedicine and vision screening, ^73– 81^ while four studies utilised a quantitative clinical study.

The application of interventional approaches was noted in six studies. ^82– 87^ Additionally, three studies were experimental. ^88– 90^ Included were also two quantitative feasibility studies ^91, 92^ and four descriptive studies. ^93– 96^ Furthermore, three studies were community-based research, ^97– 99^ and two were conducted as nationwide or national surveys. ^100, 101^ Two studies which utilised a qualitative approach ^102, 103^ and one as a pilot study approach ^104^ were included. The sample sizes ranged from 21 to 289 for qualitative studies and 53 to 1.4 million for quantitative studies.

### Methodological quality

Among the 80 studies that were assessed for quality using the MMAT 2018, 59 were assessed as quantitative, 12 as quantitative randomized controlled trial, seven as quantitative non-randomized studies and two as qualitative. The quality appraisal of the included studies using the MMAT 2018 tool revealed several consistent strengths and a few common limitations across the evaluated studies. All studies had well-defined research questions and collected data that was appropriate for answering them. The samples were representative of their respective target populations, and the sampling techniques used were relevant for every study. Additionally, a strong approach to data collecting and construct validity was demonstrated by the measurements employed to evaluate results, which were consistently adequate and suitable for qualitative research as well as descriptive, controlled trials. This demonstrates strong methodological alignment with the research aims and supports the external validity of the studies.

One significant limitation emerged concerning the risk of non-response bias. In majority of studies, it was characterized as “can’t tell,” meaning that non-response rates were either not recorded or were not sufficiently addressed. This omission raises concerns about the potential influence of selection bias and its impact on the generalizability of findings. All the studies used statistical analyses that were generally appropriate, and each study utilized methods aligned with its research questions and data type. This supports the internal validity of the findings and the credibility of reported associations or distributions. Despite the studies demonstrated methodological soundness in sampling, measurement, and analysis, the common absence of detailed reporting on nonresponse bias represents an area for improvement in future research reporting.

### Interventions

Among the included studies, 10 eye health promotion interventions were categorized as: health education, health counselling, vision screening, eye screening and training, health prevention, health promotion, health policy, ophthalmic intervention, comprehensive eye examination and screening for diabetic retinopathy. The detailed findings from included studies can be found in Table 1. ^22^ The eye health promotion intervention components were frequently underpinned by the WHO vision and eye screening implementation handbook and evidence-based guidelines. ^105– 108^

**Table 1.** Intervention summary matrix (N=80).

Intervention type	Settings	No. of studies	Professionals involved	Key outcomes	Common limitations
Health Education	Schools, Communities, Hospitals, Online	9	Ophthalmologists, Optometrists, Opticians, Ophthalmic Technicians	↑ Awareness, ↑ Spectacle compliance (in some studies), Improved parental knowledge	Lack of structured follow-up, minimal technological integration, mixed compliance rates, No monitoring, Lack of evidence-based practice
Health Counselling	Primary Care, Clinics	1	Ophthalmologists	Identified gaps in protective eyewear counselling	No monitoring/follow-up; lack of consistent messaging, Weak family involvement, limited attention to ethical standards, lack of innovation
Vision Screening	Schools, Communities, Hospitals, Mobile Units	25	Ophthalmologists, Optometrists, Orthoptists, Opticians, Ophthalmic Technicians	Early detection of refractive errors and visual impairments	Low referral completion, poor spectacle compliance, uneven follow-up, Contrast sensitivity omitted in many interventions
Eye Screening & Training	Schools	1	Ophthalmologists, Optometrists, Teachers	Increased capacity through training, improved referral rates	Limited to one country (Peru), lacking M&E framework
Health Prevention	Schools, Hospitals, Primary Care	4	Ophthalmologists, Ophthalmic Assistants	Improved DR outcomes, ↑ Screening compliance, Lifestyle behaviour change	Small sample sizes, limited long-term tracking, weak multi-sector collaboration, lack of M&E framework, minimal technological integration
Health Promotion	Schools, Communities	6	Ophthalmologists, Optometrists	↑ Eye health literacy, ↓ Myopia risk, ↓ Trachoma prevalence	Limited evaluation metrics, minimal integration with health systems, weak M&E framework
Health Policy	Communities	1	Ophthalmologists, Optometrists	Identified access gaps, workforce shortages	No implementation outcomes reported, no technological us, weak multi-sector collaboration, weak M&E framework
Ophthalmic Intervention Program	Retirement homes, Communities, Hospitals, Workplaces	11	Ophthalmologists, Optometrists, Ophthalmic Technicians, Opticians	↑ Access to care, ↑ DR detection, ↑ Follow-up rates, mHealth success	Variability in impact, low adherence in elderly, lack of cost data, weak M&E framework, Limited behaviour change and skill development
Comprehensive Eye Exams	Schools, Retirement Homes, Communities	6	Optometrists, Ophthalmologists, Refractionists	Identified multiple eye conditions, some ↑ in compliance, impact on myopia risk	No intraocular pressure, visual fields, pupil examination or dry eye exams included, low spectacle adherence
Diabetic Retinopathy Screening	Primary Care, Hospitals, Communities	14	Ophthalmologists, Optometrists, Ophthalmic Assistants, Ophthalmic Nurses, Orthoptists	↑ DR detection, ↑ Screening uptake, Telemedicine feasibility	Participation gaps in LMICs, variable guideline adherence, limited education components

- ↑ = Increased/Improved - DR = Diabetic Retinopathy - M&E = Monitoring and Evaluation - LMICs = Low- and Middle-Income Countries.

### Health education (Developing Personal Skills)

The health education interventions in this review were from nine studies and focused on improving spectacle compliance, parental awareness of paediatric eye conditions, and patient education. Two studies used schools, ^64, 89^ three used hospitals ^30, 67, 90^ and four used community-based approaches for health promotion intervention. ^63, 87, 95, 102^ The interventions were delivered by optometrists, opticians and the majority being ophthalmologists ^30, 67, 90, 102^ emphasizing the role of eye care professionals in promoting vision health. The sample sizes varied, with some studies involving large populations (e.g., 15,404 participants, ^64^ while others had smaller groups (e.g., 21 participants), ^102^ and a combined population sample of 24 892. ^30, 63, 64, 67, 87, 89, 90, 95, 102^ Health-related issues addressed primarily focused on education related to spectacle use and compliance, parental awareness of paediatric eye conditions, and online patient education. All the studies were population-centred, preventative, empowering, supportive and community-engaging. Only one study utilised online education suggesting a need for innovation and technology in health education. ^102^ There were no differences in interventions from studies conducted in high-income countries (HICs), ^30, 63, 67, 87, 95, 102^ upper-middle-income countries (UMICs) ^64^ and (LMICs). ^89^

Notably, one study found that online patient education significantly improved knowledge of eye health, highlighting the impactful potential of digital learning tools. ^102^ However, one study identified a gap in parental knowledge regarding paediatric vision care, suggesting the need for more targeted awareness campaigns. ^95^ The study that tested educational interventions promoting spectacle use found no significant difference in purchase rates, ^64^ while another demonstrated a significant improvement in spectacle compliance following a structured educational intervention. ^89^ A significant improvement in follow-up compliance after educational intervention was observed. ^30, 63, 87, 89, 90^ Overall, the effectiveness of these interventions varied, with most studies demonstrating clear improvements in eye health awareness and behavioural change, while a few showed limited impact, emphasizing the need for continuous evaluation and adaptation of health education strategies. All studies were limited in their interventions, with a lack of structured monitoring and evaluation, which is crucial for assessing the long-term impact of health education initiatives.

### Health counselling (Developing Personal Skills)

The health counselling intervention in one included study aimed to address protective eyewear use, patient education, and behavioural counselling in monocular patients. One study from a HIC investigated counselling practices related to protective eyewear for monocular patients. ^32^ A study with a sample of 60 participants highlighted gaps in health counselling practices, and the need for structured and consistent patient education strategies in primary care settings. Ophthalmologists were the eye healthcare professionals involved in this intervention. ^32^ The findings showed that 56.7% of patients did not recall receiving counselling, while 35% had received advice, and 8.3% remained unsure of any counselling provided. ^32^

Despite the potential benefits of health counselling, lack of continuous monitoring and follow-up were flagged as key limitations of these interventions. The intervention in this study had behavioural change strategies, evidence-based approaches, and preventive counselling integrated however community and family participation, technological innovation, and emotional support were lacking. ^32^ Additionally, ethical and confidential practices were not addressed.

### Vision screening (Creating Supportive Environments)

A total of 25 studies had vision screening interventions, mostly aimed at identifying various ocular conditions across diverse populations. The vision screenings were conducted in schools, ^31, 35, 38, 41, 45, 46, 51, 53, 58, 62, 77, 81, 93^ within communities, ^25, 26, 55, 57, 66, 71, 72, 91, 94, 104^ and hospital settings, ^49, 80, 101^ and were aimed at improving early detection and timely intervention. The interventions involved optometrists, ophthalmologists, orthoptists, and opticians, demonstrating a multidisciplinary approach to vision screening. Sample sizes ranged from 113 participants ^91^ in a study evaluating tele-ophthalmology for remote screening, to 1.4 million, ^84^ in a study assessing national vision screening coverage.

These studies highlight the importance of large-scale screening efforts in detecting and managing vision problems early. The key health issues addressed by vision screening were possible refractive errors and ocular conditions in children. In conducting the vision screening, visual acuity was assessed in all studies, while refractive error screening was evaluated in 19 studies ^26, 35, 38, 41, 46, 49, 51, 53, 55, 57, 58, 66, 71, 72, 77, 91, 93, 94, 104^ and binocular alignment assessed in 11 studies. ^26, 35, 38, 41, 45, 46, 55, 57, 58, 77, 94^ Other studies also screened for colour vision, ^38, 46, 58, 81^ intraocular pressure, ^25, 38, 49, 57, 66, 71, 91, 93, 104^ intraocular health, ^25, 35, 41, 46, 49, 55, 57, 66, 71, 77, 80, 91, 93, 94, 101, 104^ contrast sensitivity ^71, 93^ and visual fields. ^25, 71, 80, 81, 91^ Most of the vision screening interventions were conducted in high-income countries (HICs), a few in UMICs ^35, 41, 51, 93^ and only two ^31, 62^ in LMICs.

One study assessed the spectacles cut-offs for providing spectacle in a school-based vision screening suggesting that children with ≥-0.75D myopia, >+1.00D hyperopia, and >+0.75D astigmatism had significantly greater improvements in vision than those with lower refractive errors. ^51^ However, during follow-up, there were only 31% of children wearing their spectacles. Similarly, an assessment of the Self-Assisted Vision Examination (SAVE) method found that 66% of the children were referred for refraction, and 81.3% of those who received glasses were wearing them at follow-up, indicating high compliance. ^62^ Another study reported that 53% of referred children from the vision screening had visual impairment eligible for spectacle correction. ^45^ These findings suggest that while school-based screening improves detection, spectacle compliance remains a challenge. ^45, 51, 62^ Community-based screening programs were effective in increasing early detection of ocular diseases; however, challenges related to follow-ups and referrals were noted. Two USA-based studies identified inefficiencies in traditional vision screening programs, associated with over-referrals and poor follow-ups. ^53, 104^ Another found that 83% of individuals attended their optometric exams following a community screening, with 70.5% being referred to ophthalmology for further evaluation. ^66^ These studies emphasized the importance of integrating structured referral pathways and follow-up mechanisms into community-based vision screening interventions. ^53, 66, 104^

Remote vision screening using teleophthalmology has been identified as feasible and effective for detecting retinopathy and other eye diseases in rural areas. ^49, 91^ Approximately 74.7% of neonates underwent vision screening, emphasizing broad national-level coverage. ^101^ A study assessing preschool vision screening found that 65% of referred children attended follow-up eye exams, with 74% of those examined prescribed spectacles. ^38^ Meanwhile, the Vision First Check Program increased preschool participation in vision care, reinforcing the need for integrated screening programs at early childhood levels. ^38, 94, 101^

A study on mobile ophthalmic unit screenings reported that 88.5% of diagnoses from mobile screenings matched tertiary hospital evaluations, validating their accuracy and effectiveness. ^57^ Nearly 100% of school screening coverage, with 9-15% of screened children requiring referrals, demonstrated high screening reach coupled with referral inefficiencies. ^25, 57, 58^ Screening for vision impairment among elderly populations revealed high prevalence rates but limited post-screening interventions. ^25, 80^ A 38% prevalence of visual impairment reported in nursing home residents underscores high unmet needs in elderly eye care. ^72^ The vision screening in frail older adults reported no significant improvement in visual acuity after 12 months, suggesting the need for comprehensive management beyond screening alone. ^71, 72, 80^

### Eye screening and training program (Creating Supportive Environments)

Only one ^85^ was included in the systematic review which aimed to assess the scaling up of screening training to improve the detection, management, and referral of common eye conditions. The intervention included all the training program criteria and was conducted in school settings by optometrists and ophthalmologists. This intervention involved 355 individuals who were teachers and school directors, trained in vision programs. The overall health issues addressed in this intervention aimed to equip healthcare providers, teachers, and community health workers with the necessary skills and knowledge to increase the uptake of eye health services, particularly in underserved regions in Peru. The effectiveness of the intervention was based on the successful training of 355 teachers and school directors, from 315 schools across 24 districts, and led to the expansion of vision screening services in two of the eight provinces. Training of non-eye-care professionals can significantly improve access to vision screenings in remote areas, thereby enhancing early detection and referral for comprehensive eye examinations.

### Health prevention (Strengthening Community Action)

The overall aim of the four included studies was to address lifestyle modification for diabetic retinopathy management, treatment for age-related macular degeneration, uptake for paediatric rheumatology screening, and compliance with spectacle wear. ^34, 36, 82, 83^ Three of the studies were conducted by ophthalmologists, ^34, 82, 83^ and one was conducted by ophthalmic assistants. ^36^ The health prevention interventions were conducted in schools, ^36^ primary care centres, ^82^ and hospitals, ^34, 83^ and were aimed at enhancing adherence to spectacles wear and promote early detection and management of ocular conditions. Three of the studies were conducted in HICs ^34, 82, 83^ and one in a LMIC. ^36^ The cumulative sample size across the studies in health prevention was 2,000 participants. The intervention reviewed integrated multiple strategies to enhance compliance, foster behavioural change, and ensure uptake of vision care services. Four studies ensured population-centred and inclusive approaches, preventative and proactive, evidence-based approaches, empowerment and capacity-building, behavioural change and skills development, supportive environments, and community engagement. ^34, 36, 82, 83^ Two studies involved multi-sectoral collaboration ^36, 83^; one study included innovation and technology, ^83^ whereas none of the studies explicitly included a structured monitoring and evaluation plan.

The effectiveness of the health prevention intervention was analysed based on compliance, patient behaviour change, and adherence to eye care practices. Studies assessing the impact of lifestyle interventions on the development of diabetic retinopathy in participants with impaired glucose tolerance found that microaneurysms were significantly lower in the intervention group (24%) as compared to the control group (38%) (p = 0.029), indicating that lifestyle changes had long-term metabolic benefits. ^82^ Micronutrient supplement usage as a preventative measure for advanced age-related macular degeneration was found to be 93% among participants, with 61% using the correct dosage as per Age-Related Eye Disease Studies recommendations. ^34^ The effectiveness of quality improvement interventions in improving compliance with eye screening guidelines among paediatric rheumatology patients receiving hydroxychloroquine (HCQ) was 65% of baseline compliance, which increased to 85% after the implementation of quality improvement interventions and sustained over 10 months. ^83^ Investigated spectacle compliance among students, provided with spectacles during a school screening programme, showed low compliance, with only 29.8% wearing their spectacles. ^36^ Additionally, 35% of students had lost their spectacles during the follow-up session. ^36^

### Health promotion (Strengthening Community Action)

The overall aim of the six included studies was to assess health promotion interventions aimed at improving eye health literacy, change behaviours related to vision care, prevention of myopia, and increasing access to eye services in different populations. ^44, 54, 84, 86, 88, 103^ Four of the studies were conducted by ophthalmologists, ^54, 84, 86, 88^ one by optometrists, ^103^ and the last one was conducted by a collaboration of ophthalmologists and optometrists. ^44^ The health promotion interventions were conducted in schools ^44, 86, 88^ and community-based settings ^54, 84, 103^ to address eye health literacy, prevention and awareness of eye conditions, and behavioural change towards eye health knowledge and attitudes. Three of the studies were conducted in HICs, ^54, 86, 103^ one in an UMI ^88^ and two in a LMICs. ^44, 84^ The cumulative sample size across the studies in health promotion was 3 741 participants. Six studies ensured population-centred and inclusive approaches, preventative and proactive, evidence-based approaches, empowerment and capacity-building, supportive environments, innovation and technology, and community engagement. ^44, 54, 84, 86, 88, 103^ Three studies involved multi-sectoral collaboration ^54, 84, 103^; five studies involved behavioural change and skills development, ^54, 84, 86, 88, 103^ and two had a monitoring and evaluation plan. ^84, 88^

Awareness, behavioural change, disease prevention, and eye care service accessibility were at the centre of the health promotion intervention analysis in this studies. The effectiveness of a school-based eye health promotion intervention on health literacy, attitudes, and behaviours among children revealed a 10-20% increase in good knowledge, with significant improvements in awareness of common eye diseases (p < 0.001). ^44^ Similarly, another study reported that 100% of primary school learners in the intervention group consistently wore their spectacles post-intervention contrasting the 53.3% in the control group (p<0.05). This further highlighting a significant impact on eye health behaviours and awareness. ^88^ The impact of community-based improvements in water and sanitation facilities on reducing active trachoma prevalence showed a decrease from 13.3% to 1.4% over two years after the implementation of the SAFE strategy (Surgery, Antibiotics, Facial Cleanliness, and Environmental Improvements). ^84^ Two studies investigated the relationship between outdoor activity and myopia control. ^54, 86^ One study found that for each additional hour of outdoor activity per day, the risk of developing myopia was reduced by 10% (OR = 0.90, 95% CI: 0.84–0.96, p = 0.004). ^54^ Meanwhile, another study reported that children who participated in outdoor recess had significantly lower myopia onset rates (8.41%) compared to the control group (17.65%, p < 0.001). ^86^ In exploring the barriers affecting access to eye care services in at-risk populations, a study found many participants considered eye health with vision loss and spectacle needs, neglecting the preventative aspects of eye care. ^103^

### Health policy (Building Healthy Public Policy)

The health policy intervention evaluated access to and adequacy of eye care services, and only one study addressed the health policy. ^33^ The study under review included 500 participants represented through a community-based approach. The intervention addressed limited access to eye care services and was conducted in an UMIC. Optometrists and ophthalmologists were at the centre of the service delivery. ^33^ The study took a population-centred approach and emphasized preventive, proactive, and evidence-based strategies while considering multi-sectoral collaboration, empowerment, and community engagement as integral components of effective policy interventions. ^33^ The intervention lacked innovative technology and monitoring and evaluation. Severely inadequate access to eye care services was identified, with only 38.6% of Jamaicans receiving an eye exam within the recommended three-year interval, while 43.4% had never had an eye exam. The eye care provider-to-population ratio was only 2.04 per 100,000, significantly lower than population requires, highlighting critical workforce shortages. ^33^

### Ophthalmic intervention program (Building Healthy Public Policy)

Ophthalmic intervention programs involved 11 studies. ^27, 40, 48, 50, 56, 60, 65, 68, 73, 92, 96^ The studies aimed to address key health issues such as access to routine eye care, quality of life for visually impaired individuals, disease-specific interventions, and the role of technology and mobile-based programs in improving eye care utilization. Four of the studies were conducted by ophthalmologists, ^27, 40, 50, 60^ five by both ophthalmologists and optometrists, ^48, 56, 65, 73, 96^ one by opticians, ^92^ and another one by ophthalmologists and ophthalmic technicians. ^68^ The ophthalmic intervention programs were conducted in retirement homes, ^56, 65, 96^ hospitals, ^48, 50, 60, 73^ community centres, ^27, 68^ primary care centres, ^92^ and workplace settings, ^40^ and sought to address the major ophthalmic health issues. Nine of the studies were conducted in HICs, ^27, 40, 48, 50, 56, 65, 73, 92, 96^ one in a MIC, ^68^ and one in a LMIC. ^60^ The overall sample size across the 11 studies was 10,593, with individual study sample sizes ranging from 53 to 2,530 participants. All the studies ensured population-centred and inclusive approaches, preventative and proactive, evidence-based approaches, and supportive environments. Ten studies included innovation and technology, ^27, 40, 48, 50, 60, 65, 68, 73, 92, 96^ and nine studies included both empowerment and community engagement. ^27, 40, 48, 50, 56, 60, 65, 68, 96^ Four studies involved multi-sectoral collaboration, ^40, 48, 73, 96^ two involved behavioural change ^27, 40^ and development, and one had a monitoring and evaluation plan. ^50^

In a Canadian optometrists-led program, participants in long-term care facilities and retirement homes had at least one eye condition with about 28.6% of the participants in need of specialised eye care. ^96^ A culturally specific diabetic eye screening program for African Americans detected 10% of patients in need of urgent ophthalmologic care. ^27^ Furthermore, 22% needed follow-up in 3 months, and 67% required regular eye care. ^27^ A positive predictive value of 18% has been found in a tele ophthalmology-based glaucoma screening, highlighting a high agreement between the quality of ophthalmic intervention programs conducted by optometrists and hospital technicians. ^73^ A glaucoma screening and early detection study showed 88% participation, leading to 2% glaucoma diagnosis and 3% with ocular hypertension. ^92^

A nursing home-based low-vision rehabilitation program identified 198 individuals with low vision, of whom 91 (68.9%) participated in interventions and cognitive and physical limitations were identified as barriers to the effectiveness of the rehabilitation. ^65^ Evaluated national telemedicine-based retinopathy of prematurity (ROP) screening program successfully screened 2,188 infants across 20 neonatal intensive care units (NICUs) with plans to expand to 31 NICUs. ^48^ Among them, 26% (n=566) were diagnosed with ROP. One study identified a 21% prevalence of ROP among preterm infants, reinforcing the need for enhanced neonatal eye screening in low-resource settings. ^60^ Another study demonstrated that the progression of ROP can be reduced by a targeted oxygen therapy protocol with only 23% of cases advancing beyond Stage 2 compared to the 44% found prior to protocol implementation. ^50^

The prevalence of dry eye diseases among visual display terminal workers revealed that 60% of participants are diagnosed with the diseases, and the study identified prolonged screen exposure as a risk factor. ^40^ Vision-enhancing interventions such as cataract surgery, and refractive error correction have been found to have no significant improvement in physical function and cognitive status among nursing home residents. ^56^ In one study, community-based and mobile health interventions reported higher effectiveness in eye care utilization over conventional screening methods. ^68^ Furthermore, patients referred via mHealth were 1.7 times more likely to utilize eye care services as opposed to conventional screening referrals (OR: 1.7, 95% CI: 1.2–2.4). ^68^

### Comprehensive eye examination (Reorienting Health Services)

Comprehensive vision examinations were conducted in six studies. ^37, 59, 69, 70, 98, 100^ The studies aimed to address key health issues such as refractive errors in adult and child myopia prevention, and compliance with spectacles wear. Four of the studies were conducted by optometrists, ^59, 70, 98, 100^ one by refractionists ^69^ and one by both ophthalmologists and optometrists. ^37^ These comprehensive vision examinations were conducted in schools, ^37, 59, 69, 100^ retirement homes ^70^ and community-based settings. ^98^ Three of the studies were conducted in HICs ^70, 98, 100^ and the other three in UMICs. ^37, 59, 69^ The included studies had a cumulative sample size of 10,496 participants. All the studies included visual acuity in their examination. ^37, 59, 69, 70, 98, 100^ Five studies included refraction, ^37, 59, 69, 98, 100^ two studies ^37, 100^ binocular and eye coordination testing and only one study included patient history. ^100^ Eye health was included in two studies ^37, 100^ similar to follow-up and referral. ^59, 69^ Only one study included both colour vision and patient education in their examinations. ^100^ Notably, no studies included intraocular pressure evaluation, pupil examination and dry eye assessment as part of the vision examination.

Comprehensive vision screening programs in school settings were effective in identifying refractive errors, amblyopia, and other eye conditions. One study conducted a mandatory eye examination study among children entering the public school system, revealing that 740 children had spectacles prescribed, 181 were diagnosed with amblyopia, and 123 were diagnosed with strabismus. ^100^ Similarly, in two studies investigating the outcomes of free spectacle provision, one study showed a low compliance rate of 13.4%, despite the provision of free spectacles ^59^ and a randomised control study show no evidence that receiving free eyeglasses worsened uncorrected visual acuity among middle school students. ^69^

Studies evaluating outdoor activity as a preventative measure for myopia consistently found a significant effect. Analysing the association between outdoor time and myopia onset in children aged 3-9 years found that greater time spent outdoors during these ages reduced the risk of developing myopia in later childhood. ^98^ The most protective effect was observed at age nine, with a hazard ratio (HR) of 0.86 (95% CI: 0.78–0.93, p = 0.001) per standard deviation increase in outdoor time per day. ^98^ One study which examined the impact of outdoor activity and other factors on myopia progression among primary school students found that longer axial length and myopia progression were a result of less outdoor activity, more indoor studying, maternal myopia, and urban residence. ^37^

### Screening for diabetic retinopathy (Reorienting Health Services)

Fourteen studies were included in the screening for diabetic retinopathy intervention. Overall the studies sought to establish the efficacy of diabetic retinopathy screening interventions in improving early detection, improving patient uptake, and integrating screening within broader diabetes management systems. ^28, 29, 39, 42, 43, 47, 52, 61, 74– 76, 78, 97, 99^ Eleven studies involved ophthalmologists, ^28, 29, 42, 47, 52, 74– 76, 78, 97, 99^ four involved optometrists ^43, 61, 76, 97^ and three involved orthoptists. ^29, 52, 78^ Additionally, two studies involved ophthalmic assistants ^39, 99^ and one involved ophthalmic nurses. ^75^ Five studies were conducted in primary care centres, ^29, 47, 61, 78, 99^ five in community-based settings ^28, 39, 43, 52, 76^ and four in hospitals. ^42, 74, 75, 97^ Ten of the studies were conducted in HICs, ^28, 29, 42, 47, 52, 61, 74, 75, 78, 97^ three in LMICs ^39, 43, 99^ and one was from UMIC. ^76^ Across the 14 selected studies reviewed, individual study sample sizes ranged from 95 to 782,799 participants, demonstrating a wide range of implementation scales. All the studies targeted diabetic patients to ensure early detection for the prevention of progressive diabetic retinopathy. All the studies reported using non-invasive screening methods and only 13 studies detected early signs of diabetic retinopathy. ^28, 29, 39, 42, 47, 52, 61, 74– 76, 78, 97, 99^ Risk assessment for progression was included in 12 studies ^28, 29, 39, 43, 47, 52, 61, 75, 76, 78, 97, 99^; nine adhered to screening guidelines, ^28, 29, 42, 74– 76, 78, 97, 99^ and five included patient education and counselling in their screening. ^29, 52, 75, 78, 99^ Seven studies followed a structured follow-up system ^29, 43, 47, 52, 75, 97, 99^ and ten considered cost-effective and accessible screening, ^39, 42, 43, 47, 52, 61, 75, 78, 97, 99^ with 13 integrating the screening for diabetic retinopathy with broader diabetes care. ^28, 39, 42, 43, 47, 52, 61, 74– 76, 78, 97, 99^

Studies showed that intervention from early detection significantly enhanced diabetic retinopathy (DR) identification rates with 39% of screened diabetic patients having DR, 9.8% having sight-threatening DR, and 5.5% having visual impairment. ^76^ Similarly, another study reported that 33.1% of screened patients had suspected DR, while 65.3% showed no signs of disease. ^42^ Two studies reporting on telemedicine demonstrated web-based screening in primary care effective at 89.6% of retinal images gradable and 22.7% prevalence of DR detected through teleophthalmology screening. ^47, 76^ Another study found that digital fundus photography had 90% sensitivity in detecting severe DR. ^74^

Two studies reporting on mobile screening programs for DR reported effectiveness particularly in rural and underserved areas where 70.25% of patients reported not having seen an ophthalmologist in over two years whilst 33% had received eye care annually. ^29, 52^ Of the studies that focused on community-based programs, significant progress was observed in detecting DR however, only 32% of eligible participants in one study participating in the screening for DR due to lack of awareness. ^39, 43^ In one study, the effectiveness of nurse-led DR screening clinics achieved a 92% success rate with ophthalmologists. ^75^

## Discussion

This review aimed to evaluate the nature and effectiveness of eye health promotion interventions implemented in various multi-sectoral settings. This review collected heterogeneous studies presenting evidence in the implementation of eye health promotion interventions among various eye health professionals. Most studies showed a significant impact of eye health promotion interventions. The overall studies demonstrate a diverse range of strategies that have shown significant effectiveness across various settings.

Understanding the mechanisms of intervention success in eye health promotion requires an integration of public health, behavioural science, and health system theories. ^109^ The Ottawa Charter for Health Promotion served as the primary framework in this review. It outlined the five areas of health promotion, namely: developing personal skills, creating supportive environments, strengthening community action, building healthy public policy, and reorienting health services that underpin the intervention in this review. ^110^ Each intervention aligns with at least one Ottawa Charter principle suggesting a comprehensive approach to health promotion. However, many studies in this review lacked structured monitoring and evaluation, which compromised the ability to assess long-term impact.

Health education and counselling interventions were effective in improving eye health literacy and awareness, particularly among students, parents, and patients with vision conditions. Studies highlighted the importance of early education on spectacle compliance, paediatric vision conditions, and online patient education. ^30, 63, 64, 67, 87, 89, 90, 95, 102^ Similarly, community eye-health education interventions demonstrated improved eye-health knowledge retention, with scores increasing from 71.2% to 97.2% after education sessions the Vision Detroit Project. ^111^ According to a study conducted in Vietnam, eye health promotion in schools raised students’ understanding of eye health by 10-20%, and after the intervention, eye examination uptake increased from 63.3% to 84.7%. ^44^ Notably, digital health education ^102^ demonstrated significant improvements in eye health awareness, aligning with global trends towards telehealth education models. ^112, 113^ However, behavioural adherence to spectacle compliance remained low, despite educational interventions. ^64, 89^ The lower maternal education levels have been linked to non-compliance, as mothers with limited education may not fully understand the importance of spectacle wear by several studies. ^114, 115^ Similarly, health counselling for monocular patients ^32^ revealed major gaps in communication strategies, as 56.7% of patients did not recall receiving counselling. This finding highlights the need for structured patient engagement strategies, supported by recent evidence on shared decision-making in vision care. ^116^

The WHO has emphasized digital health interventions as a key strategy for improving health literacy, aligning with findings that online patient education improved knowledge uptake. ^117^ However, low spectacle compliance suggests that knowledge alone is insufficient, requiring behavioural reinforcement strategies. ^118^ In line with findings from other LMIC-based interventions where digital platforms supported health promotion and self-management for medical conditions, our review found that online patient education platforms significantly improved knowledge uptake. ^119, 120^ However, to apply these successes to wider LMIC contexts, it is necessary to critically examine enduring digital barriers. Many rural and impoverished areas still have poor internet connectivity, and even where access exists, there are challenges due to digital literacy and data costs. ^121^ This indicates that although digital health interventions have a lot of potential, their effects are inevitably influenced by socioeconomic and infrastructure factors. Therefore, interventions must be flexible in order for digital health strategies to be genuinely effective. For example, they should include SMS-based messaging, low-data applications, and offline accessible whenever feasible. ^122^

Vision screening interventions were highly effective in the early detection of refractive errors, amblyopia, and ocular conditions in school, community, and hospital settings. School-based vision screenings identified significant refractive errors, yet spectacle compliance remained low. ^31, 35, 38, 41, 45, 46, 51, 53, 58, 62, 77, 81, 93^ Telemedicine-based vision screening ^49, 91^ was particularly effective in detecting retinopathy, supporting its integration into primary care models.

Barriers to the effectiveness of community and mobile-based eye screening programmes such as low adherence to follow-up appointments, ^53, 66, 104^ lack of structured referral pathways ^66^ and poor compliance with spectacle use, ^51, 62^ emerged consistently in our review. Global eye health reports emphasize early screening as a cost-effective measure for reducing vision impairment. ^123^ However, findings from this review indicate gaps in referral pathways and follow-up care, which aligns with recent studies on health system inefficiencies. ^124^ These gaps mirror broader systemic inefficiencies within health systems globally which are not unique to LMICs. Fragmented referral mechanisms and inadequate follow-up care have been highlighted in global health literature as critical points of failure that undermine the long-term impact of otherwise effective screening initiatives. ^125, 126^

Community-based programmes have shown a significant reduction in visual impairment prevalence and early detection as cost-effective measures. ^127^ But their effectiveness depends on timely referrals and ongoing treatment or corrective care. Without these, initial gains made through screening are frequently lost, particularly for disadvantaged populations that have difficulty accessing secondary or tertiary services. ^128^ Our findings support recommendations for primary health care (PHC) to integrate eye health services, a strategy that has worked well in other areas of public health. For instances, PHC workers can effectively provide complex care and guarantee service continuity with the right training, support, and referral infrastructure, as shown by task-shifting strategies and integrated care models in HIV/AIDS and TB programmes. ^129, 130^ These models demonstrate how, with the right mobilization of human and logistical resources, utilizing the frameworks of the current health system can enhance the efficiency and accessibility of eye care.

Health prevention and promotion interventions were effective in influencing lifestyle modifications, disease prevention, and myopia control. ^34, 36, 82, 83, 44, 54, 84, 86, 88, 103^ The prevention of myopia through increased outdoor activity ^54, 86^ reduced myopia risk by 10% per additional hour spent outdoors. In addition, trachoma prevalence decreased from 13.3% to 1.4% following the implementation of the SAFE strategy as a health promotion intervention. ^84^ These results align with recent findings from a systematic review which confirm outdoor activity’s role in delaying myopia onset. ^131^ However, school-based interventions for spectacle use showed inconsistent results, emphasizing the need for school-wide enforcement policies.

One study ^33^ evaluated eye care policy gaps, revealing severe inadequacies in workforce availability, with only 38.6% of Jamaicans receiving an eye exam within the recommended period. This finding underscores systemic challenges in eye health service delivery, particularly in resource-constrained settings where infrastructure and human resources are limited. Workforce shortages in eye health have been recognized globally as a barrier to achieving effective coverage. According to the World Report on Vision one of the major contributing factors to VI is the lack of trained eye care professionals, especially in LMICs. ^6^ Outdated optometric laws governing the activities of eye care professionals compounds the problem in the Jamaican study. Recent evidence supports the urgent need to integrate primary eye care into existing health systems as a sustainable and cost-effective strategy to bridge this gap. ^132^ This model has shown success in countries like India and Rwanda, where task-shifting and upskilling of primary health workers have improved coverage and reduced avoidable blindness. ^133^ Furthermore, expanding the optometric workforce is vital to achieving these integration goals. As noted is several studies, there is a global shortage of eye health professionals, with optometrists playing a pivotal role in the early detection, management, and referral of eye conditions. ^133, 134^ Expanding training programs, establishing clear scopes of practice, and strengthening policy frameworks are necessary steps to ensure that optometrists are fully utilized within the health system.

Comprehensive eye examinations and diabetic retinopathy screening ^28, 29, 39, 42, 43, 47, 52, 61, 74– 76, 78, 97, 99^ demonstrated high detection rates (39% DR prevalence, 9.8% sight-threatening DR ^76^), successful telemedicine integration in DR screening ^47, 76^ and low participation due to lack of awareness. ^39, 43^ Diabetic retinopathy screening programs emphasize telemedicine as an efficient approach, reinforcing findings that 89.6% of digital fundus images were gradable. ^47^

### Strengths and limitations

This review has identified a number of gaps that may be considered in future studies. This review was conducted in English which may result in language bias potentially excluding relevant research published in other languages. Many interventions, especially vision screening programs lacked long-term follow-up making it difficult to assess sustained outcomes. Most studies were conducted in high-income countries, with limited research from low-and middle-income settings affecting the generalizability of findings. The integration of monitoring and evaluation into the design of intervention will strengthen evidence and increase understanding of future programme effectiveness. However, this review provides an important contribution as the first to explore the nature and effectiveness of eye health promotion interventions globally. It employed a rigorous methodological approach to critique the quality of the included studies and systematically evaluated the interventions through the lens of the Ottawa Charter for Health Promotion.

## Conclusion

This systematic review highlights the effectiveness of eye health promotion interventions, while also identifying gaps in compliance, follow-up systems, and policy integration. Findings emphasize the need for structured behavioural change interventions, expanded use of telemedicine, and workforce strengthening. Future research should focus on longitudinal impact assessments and cost-effectiveness analyses to inform policy and practice.

## Ethics and consent

This systematic review for part of a larger study. Ethical approval has been obtained from the Biomedical Research Ethical Committee at the University of KwaZulu-Natal: (BREC/00006067/2023). The protocol has been registered in the International Prospective Registry of Systematic Reviews.

## Data Availability

Figshare: Supplementary data, 10.6084/m9.figshare.29900339. ^22^ The project contains the following underlying data: PRISMA 2020 checklist (S1_Checklist_PRISMA 2020 Checklist), search strategy (S1 Table_Search Strategy), MMAT 2018 quality assessment results (S2 Table_Studies Quality Assessment Tool), characteristic of included studies (S3 Table_Study Characteristics). S1 Table_Search Strategy S1_Checklist_PRISMA 2020 Checklist Fig 1_PRISMA_2020_Flow_Diagram S2 Table_Studies Quality Assessment Tool S3 Table_Study Characteristics Data are available under the terms of the Creative Commons Attribution 4.0 International license (CC-BY 4.0).
