# Supplementary material for: Eye health promotion in multi-sectoral settings: A systematic review of intervention types and effectiveness
Source: F1000Res. 2025 Sep 18;14:934. [Version 1] doi: 10.12688/f1000research.168998.1 (PMC12825120; doi:10.12688/f1000research.168998.1)
Supplement: Peer review history

# Reviewer report: Reviewer response for version 1

**DOI:** 10.5256/f1000research.186252.r440360

**Published:** 2026-01-06

**Authors:** Dr Aminatu Abdulrahman

**Affiliations:** 1 National Eye Center Kaduna Nigeria, Kaduna, Nigeria

This systematic review on ***Eye health promotion in multi-sectoral settings: A systematic review of intervention types and effectiveness *** clearly stated the objective of the study, research questions  and the appropriate methodology which should be replicable. The conclusions are suitable though applicability may be limited to high and middle income settings. Recommendations for future research should also include lower income settings where health promotion and prevention as well as cost-effectiveness are more needed

Are the rationale for, and objectives of, the Systematic Review clearly stated?

Yes

Is the statistical analysis and its interpretation appropriate?

Not applicable

If this is a Living Systematic Review, is the ‘living’ method appropriate and is the search schedule clearly defined and justified? (‘Living Systematic Review’ or a variation of this term should be included in the title.)

Yes

Are sufficient details of the methods and analysis provided to allow replication by others?

Yes

Are the conclusions drawn adequately supported by the results presented in the review?

Yes

Reviewer Expertise:

public health for eye care, paediatric ophthalmology, ophthalmic anaesthesia

I confirm that I have read this submission and believe that I have an appropriate level of expertise to confirm that it is of an acceptable scientific standard.

# Reviewer report: Reviewer response for version 1

**DOI:** 10.5256/f1000research.186252.r419762

**Published:** 2025-10-31

**Authors:** Antor Odu Ndep

**Affiliations:** 1 PUBLIC HEALTH, HAVILLA UNIVERSITY, NDE-IKOM, CROSS RIVER STATE, Nigeria; 2 University of Calabar, Calabar, Cross River, Nigeria

Summary: This is a systematic review of eye health promotion interventions implemented by eye health professionals with the aim of determining the nature and effectiveness of such interventions. It followed a conventional systematic review format using CRD guidelines and reported according to PRISMA-P. Electronic databases were used including PubMed and Web of Science. The search strategy included the use of MeSH and free text terms. Data extraction followed the TIDieR. The protocol is registered with PROSPERO and published in PLOS one. The health interventions were health education, health counselling, vision screening, health prevention, health policy, ophthalmic intervention program, and screening for diabetic retinopathy. These interventions were found to have significant effectiveness across various settings.

 This is a traditional systematic review therefore statistical analysis was not applicable, and it is not a 'living' systematic review either.

Are the rationale for, and objectives of, the Systematic Review clearly stated?

Yes

Is the statistical analysis and its interpretation appropriate?

Not applicable

If this is a Living Systematic Review, is the ‘living’ method appropriate and is the search schedule clearly defined and justified? (‘Living Systematic Review’ or a variation of this term should be included in the title.)

Not applicable

Are sufficient details of the methods and analysis provided to allow replication by others?

Yes

Are the conclusions drawn adequately supported by the results presented in the review?

Yes

Reviewer Expertise:

Public health education and health promotion, maternal and child health, qualitative research

I confirm that I have read this submission and believe that I have an appropriate level of expertise to confirm that it is of an acceptable scientific standard.
